# Clinical Impact of Familial Hypercholesterolemia on Lower Extremity Artery Disease in Premature Patients

**DOI:** 10.3400/avd.oa.25-00141

**Published:** 2026-02-04

**Authors:** Eisaku Ito, Takao Ohki, Hiroshi Yoshida, Kenjiro Kaneko

**Affiliations:** 1 Division of Vascular Surgery, Shinyurigaoka General Hospital, Kawasaki, Kanagawa, Japan; 2 Division of Vascular Surgery, Department of Surgery, The Jikei University School of Medicine, Tokyo, Japan; 3 Department of Laboratory Medicine, The Jikei University Kashiwa Hospital, Kashiwa, Chiba, Japan

**Keywords:** familial hypercholesterolemia, lower extremity artery disease, chronic limb-threatening ischemia

## Abstract

**Objectives:**

Familial hypercholesterolemia (FH) accelerates systemic atherosclerosis and worsens prognosis from youth. While present in 5%–10% of premature coronary artery disease (pCAD) cases, its prevalence and impact in lower extremity artery disease (LEAD) remain unclear. This study investigated FH prevalence and prognostic impact in premature LEAD (pLEAD).

**Methods:**

We retrospectively analyzed LEAD patients aged ≤70 years undergoing first revascularization. FH was diagnosed according to the 2022 Japan Atherosclerosis Society Guidelines, based on dyslipidemia and Achilles tendon thickness. Primary outcomes were survival, amputation-free rate, and secondary intervention-free rate.

**Results:**

Among 66 pLEAD patients (median age 66 years, 76% male), 10 (15%) met the FH criteria. Compared with non-FH patients, FH patients more frequently presented with chronic limb-threatening ischemia (CLTI) (90% vs. 36%, p = 0.001), bilateral lesions (100% vs. 36%, p <0.001), and dialysis dependence (90% vs. 25%, p <0.001). Three-year survival (28% vs. 90%, p <0.001), amputation-free rate (64% vs. 89%, p = 0.028), and secondary intervention-free rate (38% vs. 63%, p = 0.031) were significantly lower in FH patients. In the CLTI subgroup, survival was markedly reduced in FH (17% vs. 71%, p = 0.011).

**Conclusions:**

FH was present in 15% of pLEAD patients and associated with poor outcomes. Routine FH screening, including pCAD history and Achilles tendon evaluation, may improve prognosis.

## Introduction

Familial hypercholesterolemia (FH) is an autosomal dominant disorder of low-density lipoprotein (LDL) metabolism that leads to accelerated systemic atherosclerosis from a young age, increasing risks of coronary artery disease (CAD), carotid stenosis, and lower extremity artery disease (LEAD).^1–11)^ Its prevalence is approximately 1 in 150–300 individuals, accounting for an estimated 14 million patients worldwide.^2,3,12)^ The results of the meta-analysis showed that FH is found in 5%–10% of premature CAD (pCAD) cases, prompting routine family history and Achilles tendon evaluation in clinical practice for CAD.^2,3,9,13–16)^ In recent years, while awareness of FH has advanced in the field of cardiology, it has lagged in vascular medicine.^17)^ For LEAD, the odds ratio was 5–10 times higher in FH patients compared with non-FH individuals.^15)^ The prevalence of LEAD among FH patients has been reported to range from 8% to 16%, indicating that LEAD complicated by FH is not uncommon.^4,5,7)^ On the other hand, there are few reports about the impact of FH on limb and life prognosis in premature LEAD (pLEAD) patients. This study aimed to clarify FH prevalence and its prognostic impact in pLEAD patients.

## Materials and Methods

We retrospectively reviewed consecutive patients aged ≤70 years who underwent first revascularization for pLEAD at our hospital from January 2015 to December 2023. Exclusion criteria were revascularization for aortic dissection, acute limb ischemia, or vascular trauma. FH was diagnosed by 2 positive findings composed of increased Achilles tendon thickness (≥8 mm in males or ≥7.5 mm in females) measured with preoperative computed tomography (CT) imaging and evidence of dyslipidemia (either through elevated serum LDL cholesterol [LDL-C] levels with ≥180 mg/dL or on statin therapy) based on the Japan Atherosclerosis Society guidelines for prevention of atherosclerotic cardiovascular diseases 2022 because the family history was not entirely reviewed.^3,15,18,19)^ The primary outcomes were overall survival, major amputation-free rate, and secondary intervention-free rate.

### Data collection

**Fig. 1:**
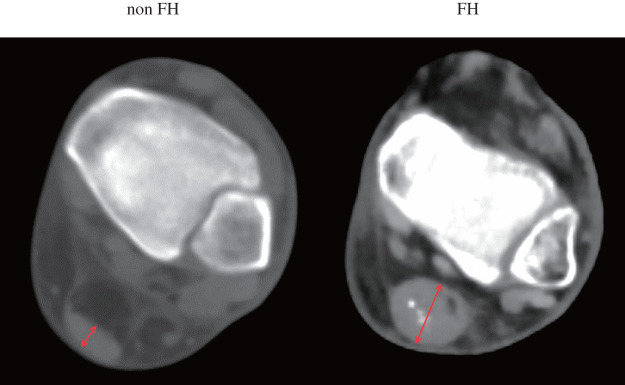
CT imaging of the Achilles tendon in patients with familial and non-FH. The red arrow highlights the measured thickness of the Achilles tendon. FH: familial hypercholesterolemia; CT: computed tomography

We extracted the respective data of patient characteristics, comorbidities, imaging, procedural details, and follow-up outcomes from the database. After axial images of CT scans were transferred to a workstation (SYNAPSE VINCENT 4.4; Fujifilm, Tokyo, Japan), CT values were calculated, and images were visually assessed using a small displayed field of view of 10 cm; the results were compared. For CT measurements, values of 1 pixel (0.195 × 0.195 mm) on the images were measured. For visual assessment, 2 radiology technicians, blinded to other aspects of the study, independently measured Achilles tendon thickness on CT short-axis images at the maximum thickness site^1,3,20–22)^ (**Fig. 1**). Each observer repeated this technique on each CT scan, and the mean score was used.

### Statistical analysis

All statistical analyses were performed using Stata/IC (Stata Statistical Software, version 14.0; StataCorp, College Station, TX, USA). We analyzed each of the preoperative factors using log-rank tests. Probability (p) values <0.05 were considered significant.

## Results

**Fig. 2:**
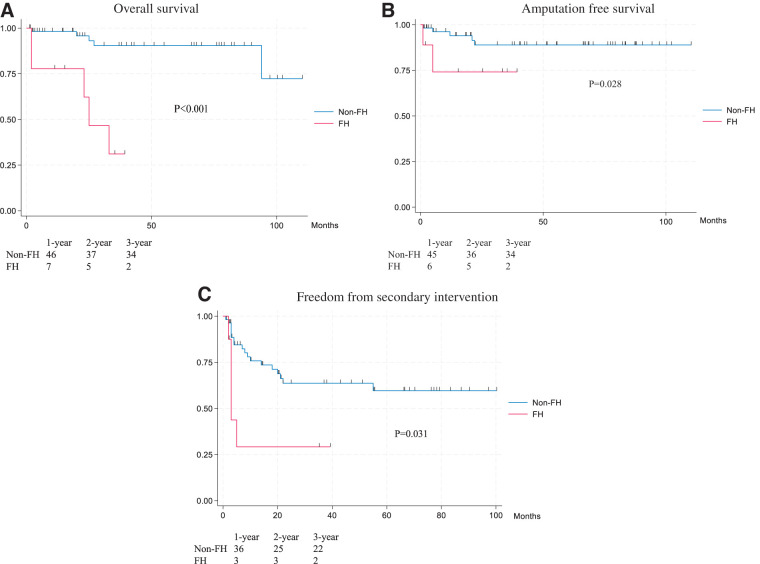
Overall survival (**A**), amputation-free survival (**B**), and freedom from secondary intervention (**C**) in patients with familial and non-FH. FH: familial hypercholesterolemia

**Fig. 3:**
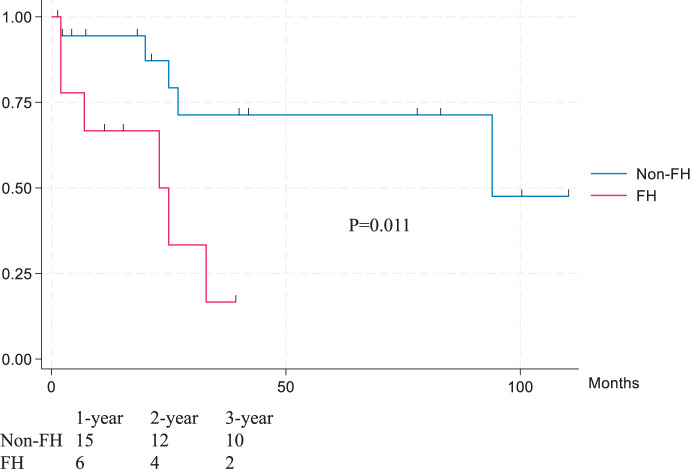
Overall survival for CLTI subgroup analysis. CLTI: chronic limb-threatening ischemia; FH: familial hypercholesterolemia

Among 66 pLEAD patients, the median age was 66 years (57–68), and 50 were males (76%); 10 patients (15%) met the FH criteria (**Table 1**). Only 2 cases were excluded because CT images were unavailable. The median Achilles tendon thickness was significantly greater in FH versus non-FH patients (10.3 mm [9.4–13.1] vs. 7.1 mm [6.3–8.1], p <0.001). The median serum LDL-C level was significantly higher in FH versus non-FH patients (159 mg/dL [110–425] vs. 94 mg/dL [45–207], p <0.001). FH cases were more likely to have chronic kidney disease (CKD) (grade 3–5) (90% vs. 50%, p = 0.019), hemodialysis (90% vs. 25%, p <0.001), and bilateral symptomatic lesions (100% vs. 36%, p <0.001). Chronic limb-threatening ischemia (CLTI: Fontaine class III or higher) was present in 90% of FH patients versus 36% of non-FH patients (p = 0.001). Three-year overall survival (28% vs. 90%, p <0.001), major amputation-free rate (64% vs. 89%, p = 0.028), and secondary intervention-free rate (FH: 38% vs. non-FH: 63%, p = 0.031) were all significantly lower in the FH group (**Fig. 2**). In the CLTI subgroup (n = 29), 3-year survival remained lower in FH than in non-FH (17% vs. 71%, p = 0.011) (**Fig. 3**). Among the 10 FH patients, 6 were on statin therapy; however, statin use did not significantly improve secondary intervention-free rate (p = 0.626), amputation-free rate (p = 0.847), or survival (p = 0.960).

**Table 1:** Clinical characteristics of patients in familial hypercholesterolemia

	Non-FH (n = 56), N (%) or median (range)	FH (n = 10)	p-Value
Age (years)	66 (62–68)	61 (57–68)	0.281**
Male	41 (73%)	9 (90%)	0.264*
Body mass index	23.2 (20.4, 24.9)	21.5 (20.0, 22.8)	0.904**
Hypertension	42 (75%)	10 (100%)	0.075*
Dyslipidemia	29 (52%)	10 (100%)	0.004*
Diabetes	29 (52%)	8 (80%)	0.098*
Chronic kidney disease	28 (50%)	9 (90%)	0.019*
Hemodialysis	14 (25%)	9 (90%)	<0.001*
Coronary artery disease	12 (21%)	10 (100%)	<0.001*
Cerebrovascular disease	12 (21%)	4 (40%)	0.207*
Serum LDL (mg/dL)	94 (45–207)	159 (110–425)	<0.001**
Fontaine			0.006*
II	36 (64%)	1 (10%)	
III	3 (5%)	1 (10%)	
IV	17 (30%)	8 (80%)	
Rutherford			0.012*
2	9 (16%)	0	
3	27 (48%)	1 (10%)	
4	3 (5%)	0	
5	14 (25%)	7 (70%)	
6	3 (5%)	2 (20%)	
CLTI	20 (36%)	9 (90%)	0.001*
ATT (mm)	7.1 (6.3–8.1)	10.3 (9.4–13.1)	<0.001**
Dutch lipid criteria	1.2 (0–8)	10.1 (9–16)	<0.001**
Bilateral lesions	20 (36%)	10 (100%)	<0.001*
Treatment			0.668*
Endovascular	48 (86%)	8 (80%)	
Surgery	6 (11%)	1 (10%)	
Hybrid	2 (4%)	1 (10%)	

* Chi-square test.

** t-test.

FH: familial hypercholesterolemia; LDL: low-density lipoprotein; CLTI: chronic limb-threatening ischemia; ATT: Achilles tendon thickness

## Discussion

In this study, 15% of pLEAD patients were found to have FH. Compared to non-FH cases, FH cases were more likely to present with CLTI, bilateral lesions, and undergo dialysis. Additionally, compared to non-FH cases, FH cases had worse prognosis, as demonstrated by higher rates of secondary intervention, major amputation, and all-cause mortality.

FH is an autosomal dominant hereditary disease known to have a poor prognosis due to systemic arteriosclerosis progression from a young age.^1–3)^ Controlling LDL-C from an early age has been shown to reduce the risk of future cardiovascular events. In a comparison between individuals diagnosed with FH in adulthood and those diagnosed early through cascade screening, the age at diagnosis was 18 years younger (39 vs. 57 years, p = 0.0001), and major adverse cardiovascular events (MACE) were significantly fewer (hazard ratio 0.67, p = 0.0044).^23)^ Therefore, the importance of early diagnosis and treatment of FH has been emphasized.^1,3,18)^ Since 5%–10% of pCAD cases are complicated with FH, it is recommended to evaluate family history and the presence of Achilles tendon thickening when treating pCAD patients.^3,9,12,13)^ However, it has been pointed out that FH awareness among physicians performing revascularization for CAD is unexpectedly low and even lower with LEAD, and many cases may be overlooked in clinical practice.^1,5)^ In Japan, the estimated number of patients with FH is 250000, and in the United States, it is 620000. However, fewer than 1% of these cases are diagnosed; therefore, physicians involved in LEAD need to raise awareness of FH.^15)^ In this study, 15% of LEAD patients in the age range ≤70 years were diagnosed with FH, suggesting the need to consider the possibility of FH when treating young patients with LEAD, similar to CAD treatment. According to a systematic review summarizing 24 studies, the reported prevalence of LEAD among patients with FH ranges from 0.3% to 60%. This substantial variability is likely attributable to the diverse diagnostic methods employed for both FH and LEAD. In contrast, this study found that the prevalence of FH among pLEAD patients who had undergone revascularization was 15%, and, to the best of our knowledge, no previous studies addressing this specific population have been identified in our search.^5)^ In the FH subgroup analysis of this study, the ameliorative effect of statin administration on prognosis was not seen. Given the limited number of cases, there may be a possibility of a statistical type II error; nevertheless, it may indicate the necessity to initiate statin therapy before the development of LEAD. To verify this point, it is necessary to confirm the statin administration period; however, the data could not be obtained in this study. Statins were not prescribed in 40% of suspected FH cases, suggesting the need to increase awareness and more aggressively consider statin use in LEAD patients.

According to the results of the JCLIMB (JAPAN Critical Limb Ischemia Database), a CLTI database study in Japan, the prognostic factors for 2-year survival included age, malignancy, low physical activity, chronic renal failure, chronic heart failure, nutritional status, and male gender.^24)^ It has been reported that the coexistence of FH and CKD increases the risk of atherosclerosis.^15)^ In this study, the prognosis of pLEAD patients who had concomitant FH was markedly poor. The high prevalence of CKD in the FH group may have influenced their poor prognosis. Among the prognostic factors, the only treatable factor is the improvement of nutritional status and possibly physical activity and cardiac function. In this study, for the first time, FH was identified as a poor prognostic factor for CLTI patients, and no similar previous studies were found. It is established that prognosis in FH can be improved by controlling LDL-C levels. Therefore, in CLTI patients with FH, early aggressive LDL-C lowering therapy has been suggested as a modifiable prognostic factor.^1,3)^ There are 2 important aspects to diagnosing FH. The first is to prevent the progression of arteriosclerosis by actively controlling LDL-C in patients diagnosed with FH. The second is that since FH is a hereditary disease, screening relatives of the target patient can enable early diagnosis, leading to primary prevention for siblings and children.^2,3,18)^ In this study, the prevalence of FH was markedly higher among patients undergoing dialysis. These findings suggest that, in the management of pLEAD patients who are receiving dialysis, vascular medicine clinicians should be aware of the coexistence of FH. Treatments for FH include smoking cessation, weight loss, pharmacotherapy, and LDL apheresis, all of which have the potential to suppress the progression of arteriosclerosis. The usefulness of starting treatment from childhood has also been proposed, and LDL-C reduction should be considered regardless of age if LDL-C levels rise. In the future, prospective studies are needed to determine whether early diagnosis of FH and LDL-C reduction in pLEAD patients can improve life and limb prognosis.

This study has several limitations. First, it is a single-center retrospective study with a limited number of cases. Second, there is no established method for evaluating the Achilles tendon with CT. In this study, we applied the standards used in X-ray examination.^3)^ Third, FH diagnosis was clinical, and genetic diagnosis could not be performed. Conducting genetic diagnosis and accumulating more cases with accurate diagnoses will be important in the future. Finally, compared with non-FH patients, patients with FH are known to have elevated lipoprotein (a) (Lp(a)) levels; however, Lp(a) was not measured in this study.^25)^ In future research, assessment of Lp(a) will be important for risk stratification.

## Conclusion

FH affects 15% of LEAD patients aged ≤70 years and is associated with adverse limb and survival outcomes. When treating pLEAD patients, clinicians should rule out FH by assessing pCAD family history and evaluating Achilles tendon thickness to optimize treatment strategy.
